# Measles Encephalitis: Towards New Therapeutics

**DOI:** 10.3390/v11111017

**Published:** 2019-11-02

**Authors:** Marion Ferren, Branka Horvat, Cyrille Mathieu

**Affiliations:** CIRI, International Center for Infectiology Research, INSERM U1111, University of Lyon, University Claude Bernard Lyon 1, CNRS, UMR5308, Ecole Normale Supérieure de Lyon, France

**Keywords:** measles virus, central nervous system, tropism, treatments

## Abstract

Measles remains a major cause of morbidity and mortality worldwide among vaccine preventable diseases. Recent decline in vaccination coverage resulted in re-emergence of measles outbreaks. Measles virus (MeV) infection causes an acute systemic disease, associated in certain cases with central nervous system (CNS) infection leading to lethal neurological disease. Early following MeV infection some patients develop acute post-infectious measles encephalitis (APME), which is not associated with direct infection of the brain. MeV can also infect the CNS and cause sub-acute sclerosing panencephalitis (SSPE) in immunocompetent people or measles inclusion-body encephalitis (MIBE) in immunocompromised patients. To date, cellular and molecular mechanisms governing CNS invasion are still poorly understood. Moreover, the known MeV entry receptors are not expressed in the CNS and how MeV enters and spreads in the brain is not fully understood. Different antiviral treatments have been tested and validated in vitro, ex vivo and in vivo, mainly in small animal models. Most treatments have high efficacy at preventing infection but their effectiveness after CNS manifestations remains to be evaluated. This review describes MeV neural infection and current most advanced therapeutic approaches potentially applicable to treat MeV CNS infection.

## 1. Measles Virus Epidemiology

Measles virus (MeV) is the etiologic agent responsible for measles disease. Humans are the only known reservoir for MeV. Despite the availability of a very efficient vaccine [1], measles remains one of the most contagious diseases with a R0 ranking from 12 to 18 [2] meaning that (in a fully susceptible population) an infected patient will on average transmit the infection to 12 to 18 individuals. This propagation rate may even increase among people with low or compromised immunity [3]. Viral transmission generally occurs from person to person through aerosols [3] and precedes onset of skin rash, making the disease even more difficult to contain. After decades of emergences mainly restricted to the poorest countries, measles has made a strong comeback and re-emerged in industrialized countries [4] where access to the vaccine was supposed to be easier. Measles killed more than 100,000 people every year [5] since 2010. In 2017, 110,000 people died from measles, mostly children under five years old [3]. Indeed, in the absence of vaccination, children are the main targets of MeV [6], although adults can be infected as well [3]. Last year, WHO documented 268,038 confirmed cases. Nevertheless, according to other estimations, there are 7 to 20 million people getting infected by measles each year [7,8].

In most developed countries measles was considered eliminated, in recent years. However the rate of vaccination decreased due to a vaccination hesitancy, and as consequence the decreased herd immunity led to large outbreaks and today measles is considered re-emerged [4,9]. This year, in many developed countries including USA and France, there is a 300% increase in reported MeV cases compared to last year [10]. Notably, 1250 cases have been reported in the USA in 2019 (from January to October) [11]. Those outbreaks confirm the re-emergence of measles, already announced by the NIAID following MeV epidemics in 2014 (CDC).

## 2. Virus

MeV belongs to the Morbillivirus genus within the *Paramyxoviridae* family and *Mononegavirales* order. This enveloped virus produces pleiomorphic viral particles with an average size ranging from 150 to 300 nm and up to 900 nm [12]. Its genome is a negative-sense, single stranded RNA of 15,894 nucleotides that encodes six structural proteins: The nucleocapsid (N) protein, the phosphoprotein (P), the matrix (M) protein, the fusion (F) protein, the haemagglutinin (H) protein, and the polymerase (large, L) protein. Two non-structural proteins, V and C are produced from the P gene [13] and mainly alter the innate immune sensing and response [14,15,16,17].

Wild type MeV strains use signaling lymphocytic activation molecule 1 (SLAMF1, also called SLAM or CD150) and nectin-4 receptors to infect target cells [18,19,20]. MeV vaccine strains use the ubiquitously expressed CD46 molecule as an additional entry receptor in vitro [21,22]. MeV entry is pH-independent and occurs directly at the cell surface [23]. However, MeV entry may also occur by endocytosis mediated by SLAM in B-lymphoblastoid cells or A549-SLAM cells [24], and through a nectin-4-mediated macropinocytosis pathway, in breast and colon cancer cell lines (MCF7, HTB-20, and DLD-1) [25]. It was also suggested that MeV Edmonston or Hallé strains could use a macropinocytosis-like pathway in non-lymphoid and lymphoid cells when SLAM and CD46 are engaged but this remains poorly documented [26,27].

**Figure 1:**
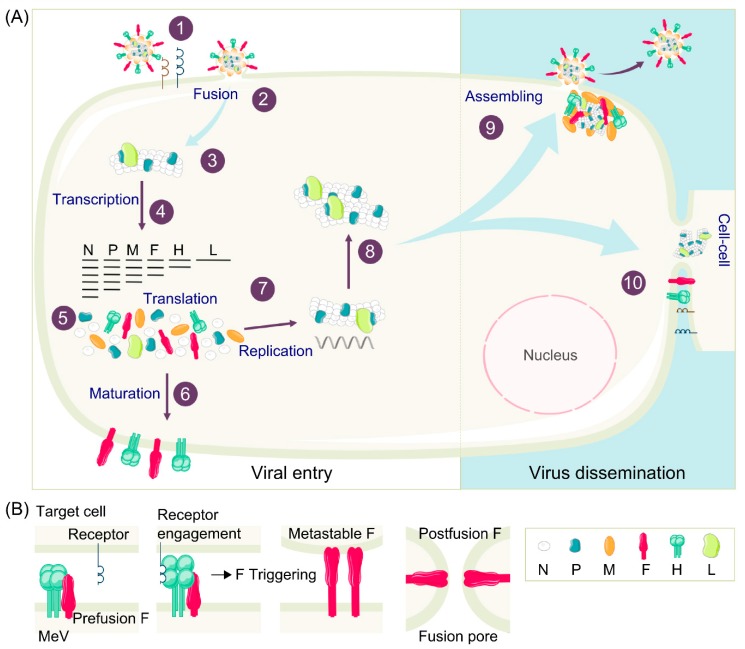
Measles Virus (MeV) replication cycle. (**A**) In order to infect a susceptible and permissive cell, MeV binds to its entry receptors on the cell surface (1) and initiates the virus-cell membrane fusion (2), as described in detail in (**B**). Virus and cell membranes fusion leads to genome delivery into the cytoplasm (3). Viral RNA is transcribed in mRNA (4) that is further translated into viral proteins (5). Viral glycoproteins maturate during their transport to the cell surface (6). The replication of positive stranded anti-genomic RNA starts in the cytoplasm (7) and serves as a template for synthesis of new negative stranded genomic RNA (8). Viral proteins assemble at the cell surface, leading either to budding of new virions (9) or cell-to-cell fusion (10). (**B**) The haemagglutinin (H) protein binds to the MeV receptor at the cell surface, allowing the triggering of fusion (F) which reaches a metastable conformation. Then, F protein anchors its fusion peptide in the target cell membrane, F undergoes serial conformational changes bringing the two membranes close enough to merge and form a pore throughout which the viral ribonucleocapsid (RNP) is delivered to the cytoplasm.

To initiate the infection of the main target cells, the MeV H protein binds to entry receptor on the surface. This attachment triggers the F protein and leads to exposure of its hydrophobic fusion peptide that then inserts into the host cell membrane. The F protein undergoes serial conformational changes allowing the merge of the host and viral membranes creating a fusion pore allowing the ribonucleocapsid (RNP) delivery in the cytoplasm (Figure 1A,B) [28,29]. Infection also spreads efficiently via cell-to-cell contact [30,31].

Transcription by the RNA-dependent RNA polymerase (RdRp) starts from a single promoter resulting in a transcriptional gradient from the most abundant mRNA for N to the least abundant mRNA for L in order to allow efficient viral cycles. These mRNAs are then translated into viral proteins. The accumulation of N and P leads to viral genome replication into positive stranded RNA anti-genome that will allow further synthesis of negative sense RNA strands that will be encapsidated by newly synthesized N, P, and L proteins [32]. Viral RNA synthesis and assembly are regulated through the interaction between M and N [33]. Viral proteins assemble to the plasma membrane and the budding of new virions can occur (Figure 1A). Alternatively, the surface glycoproteins are transported to the plasma membrane and allow cell-to-cell dissemination.

The viral RNA is encapsidated by the protein N and forms the helical nucleocapsid [34]. Each N protein covers six nucleotides, hence the genome length has to follow the “rule of 6” for being fully protected [35,36]. Together, the proteins L and P form the viral RdRp. That polymerase interacts with the nucleocapsid to progress on the viral RNA: Altogether they form the RNP.

The M protein generally ensures the viral particle integrity. The M protein also orchestrates the viral assembly at the plasma membrane and the budding of the new infectious viral particles [23].

The H and F proteins constitute the viral fusion complex that is responsible for the viral entry into the host cell. The H protein is a tetramer organized as a dimer of dimers responsible for the binding to the entry receptor. The F protein mediates the fusion between the virus and the host plasma membranes. The F is a trimer first produced as a precursor F0 that is cleaved in the trans-Golgi by a furin protease in F1 plus F2 subunits linked by a disulfide bond. The extracellular domain is constituted by the F1 and F2 subunits containing the fusion peptide at the N terminus followed by two complementary heptad repeat domains, respectively at the N terminus (HRN) and the C terminus (HRC). While the crystal structure of the prefusion form of the F protein has been described [37], the exact delimitations of the F sub-domains are still not completely defined [38,39,40].

Based on bioinformatic tools the HRN domain encompasses residues 116/138 to residue 190 and the HRC domain is included between residues 438 and 488/489. The current crystal structure however shows the region between 438 and 458 as disorganized while a canonical heptad repeat is shown after residue 458 [41,42].

## 3. Vaccines

A highly efficient live-attenuated virus vaccine is available to prevent measles outbreaks. MeV transmissibility is very high and 95% of the population needs to possess anti-measles immunity for disease eradication [43]. In 1997, during a meeting co-sponsored by the World Health Organization (WHO), the Pan American Health Organization (PAHO), and the Centers for Disease Control (CDC), the experts agreed that measles eradication was technically feasible by 2005–2010. Nevertheless, vaccination coverage decreased and led to a re-emergence of measles infection. Nowadays, measles global eradication is one of the top priorities of the expanded program on immunization (EPI) supported by the WHO. The Global Vaccine Action Plan aims to eliminate measles in five WHO Regions by 2020. Based on confirmed cases reported by the WHO, the countries with the most measles cases in 2018 were India, Ukraine, Philippines, Brazil, and Yemen. Recently, measles strongly re-emerged in industrialized countries due to the significant decrease in vaccination coverage [4,44].

Different MeV strains have been used for vaccine purpose starting with the Edmonston strain isolated in 1954 that was very reactogenic. Five vaccines were derived from Edmonston: Edmonston-Zagreb, AIK-C, Moraten, Schwarz, and Edmonston-B [45]. Some of them such as Edmonston-B remained too reactogenic. The Edmonston vaccine was replaced by the more attenuated Schwarz vaccine strain in early 60s and Moraten vaccine strains in 1968. Years later, studies have shown that Schwarz and Moraten were in fact the same virus [45]. Other vaccines derived from other strains have also been developed. Leningrad strain (isolated in 1957) attenuation successively led to Leningrad 4 and more recently to the Chinese vaccine Changchung-47. Shanghai isolate (1960) attenuation allowed production of shanghai-191 vaccine while Cam-70 which was currently produced and used in Indonesia and Japan, derived from the Tanabe (Japan, 1968) strain. All vaccines strains belong to the measles virus genotype A [45]. Measles vaccine is usually combined with mumps and rubella vaccines, known as MMR (Measles, Mumps, and Rubella) vaccine, or with mumps, rubella, and varicella (chickenpox) vaccines, called MMRV (Measles, Mumps, Rubella, and Varicella) vaccine. MMR is a live-attenuated measles virus [46]. MMR vaccination is given in a two-dose schedule, with a first dose generally administered to 12–15 months old children, and a second one three to five years later [4]. While MMR vaccine cannot be used in immunocompromised patients (with low CD4+ cell count, or severely immunedepressed), the WHO strongly recommends the vaccination of human immunodeficiency virus (HIV) positive patients without severe immunosuppression [47].

Generally, vaccinated people develop a strong humoral and cellular immunity. Only 2–10% of people who received the two vaccine doses do not produce protective measles antibodies. However, most of them remain protected by their T cell immunity [48,49].

Taken together, the too low vaccination coverage combined with the increasing proportion of immunocompromised and other non-vaccinable people call for the development of an efficient, preventive, and/or curative treatment.

## 4. Disease/Generalities

### 4.1. Symptoms and Complications

During the acute phase of MeV infection, the patients develop several symptoms, including fever, cough, nasal congestion, characteristic erythematous maculopapular rash, conjunctivitis, and pathognomonic Koplik spots on oral mucosa. Diarrhea and vomiting are often observed in infected children during the disease [50,51] or appear as a complication following the disease [5,52]. Additionally, MeV infection leads to a strong immunosuppression that can last for several months and lead to severe secondary infections [53,54]. Moreover, MeV seems to impact FoxP3 T regulatory cells homeostasis by increasing their frequency and attenuating the hypersensitivity cellular response [55]. A more recent study suggests a MeV-induced immune amnesia relying on the depletion of pre-existing memory lymphocytes [50].

**Figure 2:**
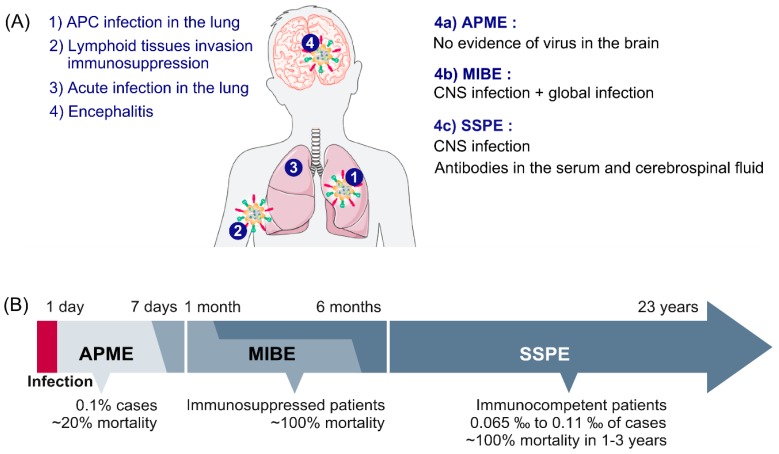
Course of MeV infection leading to measles encephalitis. (**A**) Initially, MeV infects myeloid cells in the respiratory tract. Then, MeV-infected lymphocytes disseminate the infection via the lymphatic and vascular systems. As a consequence of transient immunosuppression or autoimmunity, patients can develop acute post-infectious measles encephalitis (APME) shortly after exposure without systematic central nervous system (CNS) infection. However, measles inclusion-body encephalitis (MIBE) and subacute sclerosing panencephalitis (SSPE) are associated with MeV infection of the CNS. (**B**) The occurrence of MeV encephalitis may range from one day to 15 years following initial infection.

MeV infection can lead to several complications such as pneumonia, which is the main cause of measles mortality [56] or to central nervous system (CNS) complications, and to a lower extent to thrombocytopenia, blindness, or hearing loss [57]. Briefly, interstitial pneumonitis associated with mucosal inflammation due to large syncytia formations in the lungs are mainly observed in immunocompromised patients (Hecht’s pneumonia) [56,58]. This cytopathic effect leads to bronchio-epithelial destruction generally resolved within few days of hospitalization (Figure 2A).

### 4.2. Associated Factors (Age/Nutrition)

Multiple factors such as malnutrition and vitamin A deficiency seem to increase measles associated morbidity and mortality. Indeed, regardless of vaccination coverage, MeV-infected people in poorest countries are more likely to develop complications leading to severe disease [3,59,60,61].

### 4.3. Pathogenesis

Pathogenesis starts with MeV infection of myeloid cells in the respiratory tract. As mentioned in Section 2, the two known entry receptors for MeV wild-type strains are SLAM/CD150 and nectin-4 [18,19]. Wild-type (wt) viruses generally target lung resident macrophages and/or dendritic cells, expressing SLAM [62,63,64]. These antigen presenting cells (APCs) migrate to the lymph nodes and transmit the viral infection to SLAM expressing lymphocytes with subsequent spread of the virus in the lymphatic and vascular systems (viremia). During the late stages of the infection, circulating infected immune cells that reach the respiratory tract and the skin can transmit the infection in cis to epithelial cells expressing nectin-4 on their basolateral side [20,65,66,67]. Then, the virions produced at the apical membrane can be shed into the respiratory mucus or aerosolized in the respiratory tract through coughing [68].

## 5. Disabilities and Nervous System

MeV can also cause damages to the nervous system.

### 5.1. Hearing Loss

MeV can induce hearing loss [69]. Before the introduction of mass vaccination, hearing loss was observed in 5% to 10% of measles cases in the USA. This remains highly frequent in under-developed countries where vaccination coverage is low [70]. One possible explanation is that otitis associated with measles in up to 25% of infected patients could cause hearing loss [71]. This pathology seems related to a super infection due to MeV-related transient immune-suppression.

Alternatively, hearing loss can occur immediately after the acute phase of the infection or later following measles acute encephalitis (described in paragraph 6.1) with typical bilateral and moderate to profound sensorineural hearing loss [69]. The mechanism associated with MeV-induced hearing loss remains unclear since neither viral antigen nor RNA have been detected in samples from the inner ear [57].

### 5.2. Blindness

Eye related symptoms such as conjunctivitis or corneal inflammation (keratitis) are commonly associated with measles [57]. Corneal complications are often more serious when superinfection (bacterial or viral) occurs during MeV-induced immune-suppression. However, there is a correlation between vitamin A deficiency and measles-induced blindness. Indeed, vitamin A deficiency is associated with severe keratitis and considerably increases the risk of xerophthalmia, corneal ulceration, and blindness [72]. This may explain why measles related blindness is more common in areas where children are already suffering of malnutrition.

Viral RNA can be detected in tear secretions [73]. In addition, human ex vivo cornea rim tissue is susceptible to MeV infection on its basolateral pole but neither syncytium formation nor released infectious particle have been found [74].

The relationship between MeV infection of ocular epithelial cells and the potential relationship with neural cell infection with cases of blindness is still unclear.

## 6. Central Nervous System (CNS) Infection

How the virus enters the CNS remains unclear since the known MeV receptors are not expressed. While its expression in the CNS seems to be only transient, nectin-4 has also been suggested to play a crucial role in MeV neuroinvasion based on observations made on closely related canine distemper virus whose neurotropism directly depends on nectin-4 specific patterns of expression [65,75,76,77]. Nectin-1 positive cells have recently been shown to be able to capture membranes and their cytoplasm from the surface of adjacent cells expressing nectin-4 at their surface via a trans-endocytosis mechanism [78]. In this context, viral RNP could transit from nectin-4 positive cells in nasal turbinate or meninges to neural cells expressing nectin-1 in olfactory bulb or brain parenchyma, respectively. The following key elements involved in the neural cell-to-cell dissemination and successful CNS invasion remain to be investigated (Figure 2A).

Three main CNS complications are associated with measles: The acute and the chronic forms, the latter being subdivided in two sub-types, the first as a measles inclusion-body encephalitis (MIBE) in immunocompromised patients and the second as a subacute sclerosing panencephalitis (SSPE) occurring in immunocompetent patients [79,80] (Figure 2B).

### 6.1. Acute Encephalitis

The acute post-infectious measles encephalitis (APME) occurs in 0.1% of measles cases, about a week following the appearance of first clinical signs. The APME is also called post-infection encephalitis (PIE), acute demyelinating encephalomyelitis, or acute disseminated encephalomyelitis.

APME is associated with 20% mortality and severe neurological sequelae, mainly in adults. Symptoms include fever, headaches, seizures, and consciousness alterations. APME is a complication associated with MeV infection that seems to be related to an auto-immune reaction against the myelin basic protein mainly expressed by oligodendrocytes [81,82,83]. APME causes CNS lesions in both white and grey matters and is characterized by brain inflammation and perivenous demyelination [68,84,85,86]. Moreover, APME is often associated with more immunological abnormalities such as high levels of IgE antibodies in the serum [87]. The binding of infected leukocytes to brain microvascular endothelial cells, or a direct infection of endothelial cells themselves in the brain may also partially contribute to this inflammatory immune reaction [88]. Overall, MeV acute encephalitis is poorly described in the recent literature. Note that there is a total lack of evidence of the virus presence in the brain parenchyma compared to that in the blood circulation. Based on the absence of virus detection in certain cases, multiple groups have suggested that the encephalitis could be caused by an autoimmune-like response [89]. While the presence of myelin basic protein (MBP) in the cerebrospinal fluid (CSF) suggests autoimmune-mediated encephalitis, oligodendrocytes viability and neurons myelination have not been explored yet [89].

### 6.2. MIBE

MIBE occurs in immunosuppressed patients ranging from three weeks to six months following wild-type MeV infection or in some rare cases after inappropriate vaccination with former vaccine strains [90,91,92]. MIBE is characterized by the presence of intracytoplasmic or intranuclear inclusion bodies composed of nucleocapsids, mainly in neurons, oligodendrocytes, and astrocytes [93,94]. Patients develop febrile focal seizures and behavior disorders before lapsing into coma. At a molecular level, mutations have sometimes been observed in the intracytoplasmic domain of MeV F protein and lead to the expression of hyperfusogenic viral phenotypes. Some mutations similar to those observed in SSPE have also been detected in the N gene and it has been hypothesized that MIBE and SSPE might be very similar, apart from the more rapid development of MIBE in immunocompromised subjects [95]. Recently, other MIBE- associated mutant viruses have also been described and present an hyperfusogenic phenotype [42,92]. Notably, the mutation L454W in the HRC domain of the F protein emerged in two patients that contracted MIBE in South Africa. This mutation confers the ability of entry without the presence of known receptor even at 25 °C. The mutation L454W leads to a highly unstable F protein potentially due to a lower interaction with the H protein which loses its protection role from random triggering of the fusion protein. This finding suggests that hyperfusogenicity of these neurotropic variants allows better viral dissemination, without the need of H binding to a high affinity receptor [37,96,97].

### 6.3. SSPE

**Figure 3:**
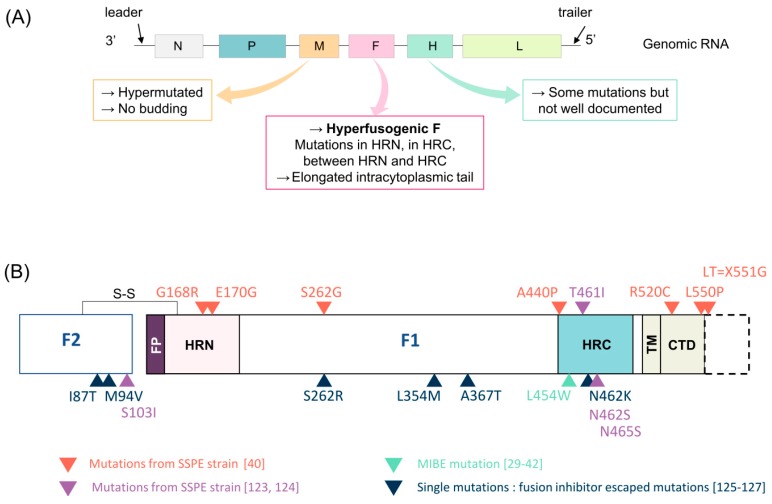
MeV F gene mutations related to CNS infection. (**A**) Schematic of MeV genome showing the most common mutations found in SSPE cases. (**B**) Details of MeV mutations in F protein leading to a hyperfusogenic phenotype and/or CNS infection.

Subacute sclerosing panencephalitis (SSPE) cases occur in 6.5 to 11 cases per 100,000 [97,98] in immunocompetent patients that contracted measles in their childhood, with a mortality rate close to 100%. Within children infected by MeV before the age of 12 months, the incidence for SSPE rises to 1/609, while reaching 1/1367 for children under five years old [99]. There is a latency period ranging from one to 15 years following primary infection and before appearance of symptoms [100,101]. In addition, because of the non-specificity of the first symptoms, SSPE diagnosis is generally delayed [102]. In most of the cases, patients do not survive more than 1–3 years following appearance of the symptoms associated with important neurological signs and dementia. Patients are developing severe physical and mental impairments but also a loss of motor control that tends to evolve in myoclonic jerks and spasms, seizures, and coma. Patients that underwent primary infection below the age of two are more at risk of developing SSPE. It was suggested that an immature immune system before two years old could contribute to persistent brain infection [83]. A dual viral hit was suggested to play a role in SSPE development. In this model, authors proposed that during classical first exposure to MeV immunocompetent patients do not develop encephalitis. However, a first exposure to a virus different from MeV, but capable of inducing an immunosuppression, which is followed with MeV infection later in the life, may favor development of CNS disease such as SSPE, as shown in the model of transgenic mice susceptible to MeV infection [103]. Most epidemiological studies are pointing that young boys are more often affected by SSPE than girls [17,104]. In one study in Germany from 2003 to 2009, the authors counted 21 males within 31 SSPE cases. SSPE is characterized by an excessive intrathecal synthesis of MeV specific antibodies. Most of the time, in the brain of SSPE patients, the genes that are encoding for MeV matrix protein (M), fusion protein (F), and attachment protein (H) are mutated [28,105,106] (Figure 3A).

## 7. Mutations Associated with MeV CNS Infection

### 7.1. M Protein

In SSPE, uridine-to-cytidine biased hypermutations of M protein are characteristic [107]. Studies have shown that MeV can evade the innate immunity control by taking advantage of the adenosine deaminase acting on RNA 1 (ADAR1), an IFN-stimulated gene that binds double-stranded RNA and converts adenosine to inosine by deamination [108]. The biased hypermutations in M (and other) gene in SSPE (or MIBE) cases might also be related to ADAR1 activity. Hypermutation of M protein leads to an unstable and defective M protein in viral particles assembly [109]. As a result, the virus is defective in budding from the plasma membranes and cannot produce viral particles. Among the large number of mutations in mRNA, the lack of the AUG initiation codon is leading to a low expression of M protein [110]. Nevertheless, in the context of brain invasion, the hypermutated M gene still allows MeV to replicate, spread, and cause disease [111,112]. Indeed, M protein negatively regulates the viral polymerase activity and thus to impact mRNA transcription and genome replication [113]. One of the roles of M protein is the distribution of both F and H glycoproteins at the apical cell surface [114]. Thereby, mutations in M protein could impact the virus fusion (and F stabilization), through association with surface glycoproteins tails, and thus influence the virus dissemination through the brain. Although in transgenic mice the infection with a M hypermutated MeV induces a more fusogenic phenotype despite attenuated budding, resulting in a more suitable virus for brain infection [111]. Other mutations impact interactions with the viral nucleocapsid and surface glycoproteins [115,116]. This provides another explanation for the absence of viral particle productions in SSPE-patient brains. This lack of budding is a key property highlighting that patients are non-contagious [93]. While numerous studies report the isolation of SSPE infectious viral particles from patient brains, none of them have physically shown whether classic infectious viral particles or virus RNP-containing apoptotic bodies expressing surface glycoproteins were effectively isolated [107,117,118,119,120].

### 7.2. F protein

The F proteins observed in SSPE cases present several mutations conferring a hyperfusogenic phenotype. F is produced as metastable protein in its pre-fusion state. This pre-fusion state is generally less stable in the CNS isolates. The F can also fuse without H engagement to any known receptor. Thus, it is suggested that these mutations facilitate CNS spread [40].

Mutations can occur in the HRC domain (T461I, A440P, N462S, N465S, and L454W), in the HRN domain (G168R/E170G), in between HRC and HRN domains (S262G), in the cytoplasmic tail domain (CTD) (R520C, L550P), and in the F2 subunit of F protein. Among the mutations found in the F-SSPE sequence from South African patient (G168R/E170G/S262G/A440P/R520C/L550P and X551G), only the mutation S262G (position already associated to hyperfusogenicity with a mutation S262R) located at the interface of three protomers, involved in fusion activation, may independently confer an hyperfusogenic phenotype to F without needing any other mutation. The functional analysis of MeV_IC323 virus carrying this F-SSPE with all seven mutations confirmed the finding that an SSPE strain can disseminate via cell-to-cell spreading in Vero cells, in the absence of known receptors [40] (Figure 3B).

The mutation of stop codon (X551G) in F-SSPE strains has been frequently observed previously [107] and leads to an elongated cytoplasmic tail (called LT for Long Tail) that can enhance the incorporation of F and nonspecific cellular protein in the virion [121,122].

Other mutations found in F extracellular domain from SSPE sequences isolated from patients brain (T461I and S103I/N462S/N465S) also confer hyperfusogenicity and can spread in human neuroblastoma cell lines and suckling hamster brains in the absence of known MeV receptors [123,124].

Fusion inhibitors such as 3G or FIP are tested on MeV and it has been documented that several mutations emerged in F protein in order to escape the treatment. The impact of these mutations (I87T, M94V, S262R, L354M, A367T, N462K) on the fusion machinery is of great interest [125,126,127]. One of the most interesting mutations that emerged is located at the residue 262. The escape mutation S262R confers hyperfusogenicity, as well as the mutant S262G that has been described in a real case of SSPE [40,123]. These data highlight the fact that emergence of mutations under a selective pressure can lead to viral adaptation to CNS. This can also allow a better design of inhibitors that could counteract these adaptation mutations.

As discussed in Section 6.2. the hyperfusogenicity correlates with a lower thermal stability of the pre-fusion state of F [28,125]. As an example, the L454W F is highly unstable and this characteristic could be sufficient to trigger F in a postfusion state by itself, allowing the fusion to occur without any receptor engagement. In the context of a circulating viral particle outside the brain, that property might not be an advantage for the virus, which could explain why any hyperfusogenic form of MeV has never been found in circulating viruses.

### 7.3. H Protein

The H protein of SSPE strains is often mutated as well and contributes to neurovirulence [128]. In a recent study, three mutations were found in the H gene of South African SSPE strain, in the cytoplasmic tail, the stalk domain, and β5 blade of the head domain, associated with substitutions R7Q, R62Q, and D530E, respectively [40]. The residue D530 is necessary for cell entry through SLAM, so the mutation D530E could compromise the use of infection through SLAM [129,130].

In a modified Edmonston strain expressing a murine-adapted H protein from a neurovirulent strain CAM/RB, the substitutions G195R and S200N lead to complete loss of neurovirulence in mice C57BL/B6 [83,131,132]. Due to questionable strains and animal model used in this study, these data have to be considered carefully and these findings might be difficult to transpose to human SSPE cases. Nevertheless, it highlights the potential existence of a specific site in H involved in neurovirulence or a site of an unknown neuron-specific receptor.

C-terminus elongation of the H protein due to single-point mutation at the stop codon have also been reported multiple times in SSPE cases [75,133]. Contrarily to deletions of the cytoplasmic tail of H which were shown to enhance fusion activity [121], elongation of the extracellular domain of H seemed to impact binding, targeting, and may explain at least partially the high level of antibodies in SSPE cases [75,133].

Unlike SSPE, mutations in H gene of MIBE virus sequences seem to be less frequent and further investigations for their potential impact in CNS infection is required [92].

### 7.4. Mutations in Other Genes

In SSPE cases, some mutations have also been found in N, P, and L proteins but most of the recent studies focused on F and M proteins. Some P genes from SSPE cases exhibit an impaired editing system that lead to less V protein production. Most of the time, the viral cycle does not seem compromised but the lower expression level of V could contribute to the viral persistence by reduced inhibition of interferon (IFN) response [134]. Is has also been shown that the P gene of the multi-mutated rodent brain-adapted strain CAMR40 is largely involved in neurovirulence, suggesting that MeV P gene could also play a role in CNS infection [135].

## 8. Animal Models for Neuro-Invasion Studies

Humans are the only natural reservoir for MeV. Thus, the choice of the best animal model remains a challenge and depends on the type of scientific questions asked, to be faithfully representative of the CNS infection in humans. A summary of the most used small animal models and their related application is presented in Table 1. Several genetically modified mice have been used, mainly to study tropism, dissemination, and to develop new treatments. Historically, the Lewis rat was commonly used to study viral tropism and dissemination through the CNS [136]. More recently, the Golden Hamsters are preferred to study MeV neurovirulence because of the similarities in the brain lesions observed by MeV in this model compared to human cases of SSPE. Moreover, unlike mice, suckling hamsters are naturally susceptible to MeV infection, especially in the brain, despite the lack of expression of any known receptors as reported in human [123,137] (Table 1). Numerous studies have been done using neurotropic strains obtained following multiple passages in Hamster brains. Nowadays, these strains, supposed to mimic persistent infection in the brain, are not used anymore. Indeed, the strains CAM/RB or HNT were highly virulent in mice, rat and hamster but the induced infection was not representative of a persistent MeV infection in the brain. These hypermutated neuro-adapted strains led to an acute infection in the brain that was not representative of the slow and progressive infection seen in SSPE [136,138,139]. Such type of infection cannot be representative of an APME or MIBE since there is no CNS infection in the first case, and there are very distinctive inclusion bodies in the brains in the second one. Nevertheless, it may allow a better understanding of the behavior of MeV once these mutations have emerged in the CNS.

Multiple murine models have been developed to address specific question about MeV pathogenesis, CNS invasion, antiviral treatments, and persistence (Table 1). Notably, MeV persistence has been demonstrated in mice infected with the Edmonston strain or a recombinant MeV expressing H from CAM/RB strain up to two months [140,141] and in nude mice with Edmonston strain highlighting the emergence of mutations [142]. SLAM transgenic (tg) ant CD46 tg mice models and derivates expressing stably and ubiquitously or not the human receptors for wt or vaccine MeV strains were also extensively used [143,144]. When these receptors are ubiquitously expressed (notably in the CNS) these very artificial models highly facilitate MeV entry. In SLAM transgenic suckling mice infected intranasally, MIBE-related mutants such as MeV F L454W were able to propagate in lungs, meninges, and neural cells in brain parenchyma confirming the maintenance of its ability to infect a host from the respiratory tract [42]. Additionally, such animal models allow not only the study of the key factors of the cells permissiveness independently of the entry step, but also to validate the efficacy of antiviral drugs in the most stringent context, since the virus spread is the most difficult to block [29,96,145,146] (Table 1).

Non-human primates represent faithful models of measles since they are fully susceptible to wild-type MeV infection [147]. Thus, rhesus and cynomolgus macaques or squirrel monkey are often used mainly for studies focusing on the acute pathogenesis [20,65,66,68,148,149,150]. These studies highlighted numerous similarities between measles pathogenesis in humans and primates. Particularly, they allowed confirming the essential role of nectin-4 for the shedding and inter-human transmission of MeV, but symptoms related to CNS infection have not been reported so far. Accidental transmission of the circulating MeV strain from human to primate have occurred notably causing five deaths out of 21 cases in rhesus monkey [151]. In this study CNS infection was not investigated and all deaths were due to secondary infection related to MeV-induced immunosuppression. In 1999, another natural outbreak led to the death of 12 Japanese macaques out of 53 cases. In the brain, demyelination was observed in one monkey and two monkeys showed neuronal inclusions with measles antigens [152] but no infectious viral particle has been isolated. In order to better characterize the CNS infection, rhesus monkeys were infected intracerebrally with a SSPE derived virus but animals did not develop any visible symptom and the virus was not detectable after three weeks, suggesting the resolution of the infection [153]. Another study reported that rhesus monkeys infected intracerebrally with hamster-brain-adapted strain developed encephalitis with morphological characteristics similar to those observed in the brain of human SSPE cases. However, as already observed in rodent, these brain infections induced by the hamster-brain-adapted strain evolved during the acute phase of infection and do not reflect the slow progression observed in the SSPE [154]. MeV CNS infection still has to be characterized in this model.

More recently, comparative analysis of MeV infection, tropism, and spread in human to canine distemper virus (CDV) in natural host species such as dog and ferrets suggested that studies of this closely related morbillivirus infection could shed light on key elements of MeV pathogenesis [155].

The tamarin (*Saguinus mystax*), often called marmoset in the literature, has been shown to be susceptible to MeV infection with Edmonston and JM strains [156,157]. The JM strain was highly pathogenic in this model, especially following cerebral inoculation [158].

## 9. MeV Tropism

Although MeV is primary a lymphotropic virus it could also infect the CNS. One of the ways the virus might enter into CNS could be through the hematogenous way by crossing the blood-brain barrier (BBB) [17]. Since endothelial cells are susceptible to infection in vitro, in vivo and in SSPE cases, their infection at the BBB could also give an opportunity for MeV to reach the CNS [88,178]. In addition, lymphocytes are also able to pass the BBB meaning that MeV-infected lymphocytes could carry the virus across the BBB [179,180]. However, the specific mechanisms allowing the virus to enter the CNS remain unclear [88,105,181]. The hyperfusogenic phenotype seems to be necessary to allow viral dissemination through neurons even in the absence of known receptor. To date, the early tropism and dissemination of *Paramyxoviridae* within the CNS during early stages of infection remain poorly documented. There are also very few available data on cellular and molecular mechanisms governing CNS invasion. To date, investigations are mainly limited to clinical symptoms, serology, RNA sequencing, and tissue immunostaining. Moreover, most of the studies have been performed with MeV vaccinal strains such as Edmonston B strain or neuro-adapted strains in Hamster, using several wild type or transgenic rodents, or other in vitro models such as primary or immortalized neural cultures. Nowadays, whether these viruses and models perfectly reflect what occurs in human remains questionable, they allow addressing specific questions in obtaining important information regarding the tropism of MeV infection in the brain.

### 9.1. Post-Mortem Studies

**Figure 4:**
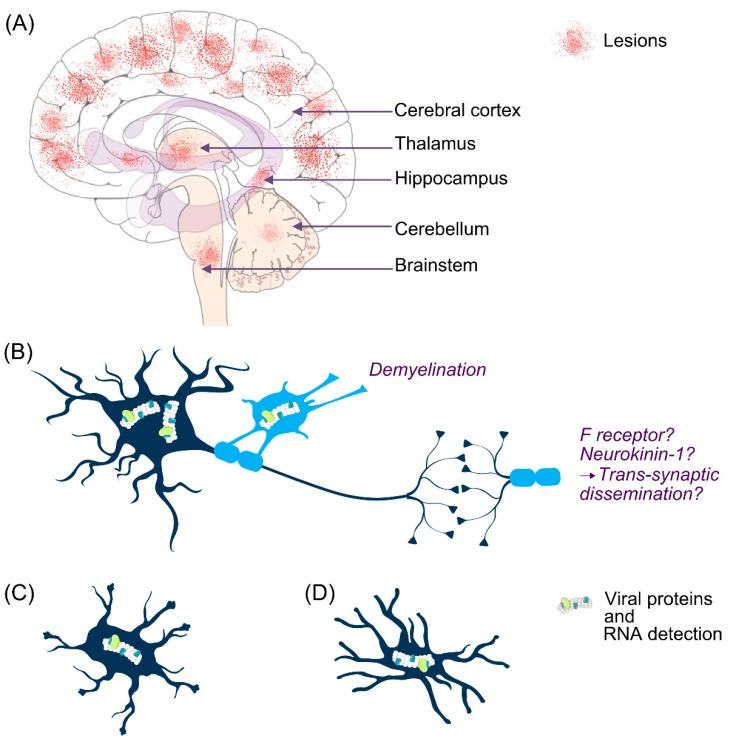
MeV central nervous system infection. Lesion areas are found in the brain of SSPE and MIBE patients but the specific areas associated with RNA detection are still poorly documented (**A**). Generally, MeV infects neurons and oligodendrocytes in humans (**B**). Occasionally, MeV RNA is also found in astrocytes (**C**) and microglia (**D**).

Post-mortem analyses of MeV-infected human brains show lesions in almost all areas (Figure 4A). In the same late context, studies of brain infection in human and animal models described the cell types harboring viral antigens in the CNS, nevertheless the early targeted and permissive cells need to be clarified.

In the CNS, MeV infection occurs mainly in neurons but also in oligodendrocytes, astrocytes, and microglia [17,182,183] (Figure 4B–D). In MIBE and in SSPE cases, viral antigens and RNA have been found in neurons and oligodendrocytes [181]. In human SSPE cases, neurons are the main target with evidence of transneuronal viral spread [97]. Infected oligodendrocytes are often located near infected neurons, suggesting oligodendrocytes infection as a secondary infection from axons. The infection of oligodendrocytes is highly related to their demyelination. The authors suggest that MeV induces demyelination that could be a hallmark of SSPE (Figure 4A,B).

Viral genome and antigen have also been found in the perinuclear cytoplasm of astrocytes, albeit with lower frequency [181].

In a study using Edmonston B strain, infection of organotypic cultures of rat hippocampus ex vivo showed that the virus can infect neurons in the absence of CD46 receptor [139].

Meninges infection has been observed following intracranial MeV inoculation in ferrets [184] and hamsters [185], as well as following intranasal infection of SLAM transgenic mice [42]. Interestingly, MeV strains and mutants used in these studies were all known as hyperfusogenic. However, meninges infection has not been reported in humans yet.

### 9.2. Early Events in MeV Infection?

It is strongly suggested that MeV may use a third receptor or co-receptor yet unknown to enter the CNS. A parallel could be done with studies of CNS invasion with the closely related CDV conducted in dog and showing that astrocytes are neither expressing SLAM nor nectin-4, but remains permissive to the infection [76].

For MeV, the hypothesis that single or combination of mutations would be sufficient to confer adaptation in brain tissues for invasion without the engagement of any receptor is also relevant. Indeed, highly unstable F mutants such as L454W, observed in MIBE cases, do not need any communication with the H for triggering and fusion and thus cell-to-cell dissemination [28]. Alternatively, there is no proof that such a virus would be able to attach to any cell in absence of H and thus go through the first event allowing the entry in the CNS. Additionally, other hyperfusogenic mutants more stable and also observed in encephalitis cases were shown to conserve there dependence on H for F triggering [40], reinforcing the idea that at least a low affinity neural receptor should allow the initial entry in a CNS cell [186].

To date, the very first cell target of MeV infection in CNS, is unknown. A recent study focused on cell susceptibility during MeV infection in the CNS using hippocampus organotypic brain cultures (OBC) from IFNAR deficient genetically modified C57BL/6 mice expressing human SLAM receptor [168]. While all cell types were susceptible to infection in the absence of IFN-I response, the permissiveness of astrocytes and microglia strongly decreased when astrogliosis was observed in immunocompetent OBC. Astrogliosis and microgliosis have been observed in MeV encephalitis [144,187,188]. These data could explain why infection of astrocytes and microglia in post-mortem analysis are barely detectable.

### 9.3. Models to Study Tropism?

The main obstacle to study early tropism of MeV and other *Paramyxoviridae* is the lack of adequate models that could faithfully represent the infection in human brains. To date, ex vivo models seem to be a good compromise [189]. Organotypic brain cultures from mice, hamsters, and rats can be generated with several brain substructures such as cerebellum, cerebral cortex, or hippocampus [139,189,190]. The advantages of this model are the presence of all four cell types in the CNS (neurons, astrocytes, oligodendrocytes, and microglia), the possibility to produce OBC from any transgenic animal, and the unique opportunity to have a direct visibility of the CNS as an open window. Moreover, several slices can be made from each substructure. Therefore, a large number of conditions can be tested with a very limited number of animals, making this model ethically preferable, compared to in vivo experiments. The main weaknesses of OBC are the lack of a vascular system with circulating leukocytes and the decreasing susceptibility to infection through time concomitant to the development of astrogliosis [168]. Murine OBC offer many possibilities but mice are not susceptible to infection so their OBC would not be suitable to study early tropism. On the other hand, golden hamsters are susceptible to MeV infection. Thus, hamster OBC might be a more relevant *ex vivo* models but the lack of tools and available antibodies for this species still strongly slows down the study of the early tropism in this model.

**Figure 5:**
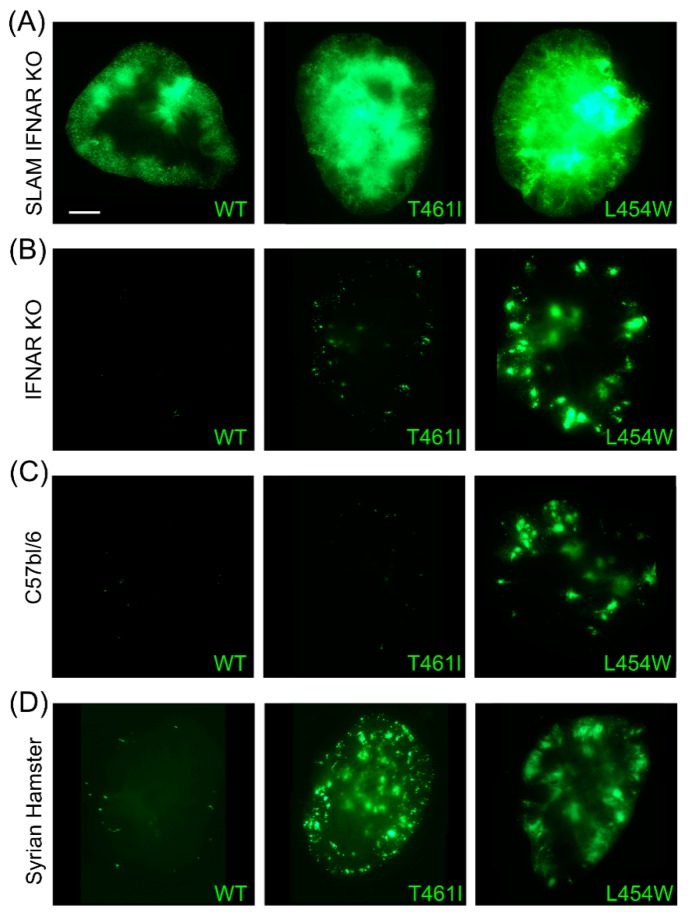
Wild-type and hyperfusogenic MeV growth in organotypic cerebellar cultures (OCC). OCC from suckling SLAM-IFNARKO mice (**A**), IFNARKO mice (**B**), wild-type C57BL/6 mice (**C**), and Syrian Hamster (**D**) were prepared as described elsewhere [189] and infected on the day of slicing with 10^3^ PFU per slice with MeV-IC323-eGFP-F-wild-type (left side images), MeV-IC323-eGFP-F-L454W (right side images) and MeV-IC323-eGFP-F-T461I (middle images). Pictures were taken at day three post infection (dpi) and reconstituted using the Stitching plug-in with ImageJ software [191]. Scale bars, 1 mm.

Organotypic cerebellar cultures (OCC) from suckling SLAM-IFNARKO mice (Figure 5A), IFNARKO mice (Figure 5B), wild-type C57BL/6 mice (Figure 5C), and Syrian Hamster (Figure 5D) allows highlighting the hyperfusogenic phenotype of MeV-IC323 bearing a L454W or T461I mutated F protein compared to the wild-type in a CNS context. The fluorescence signal is used for tracking the infection and shows the massive dissemination of the viruses MeV-IC323-eGFP-F-L454W and MeV-IC323-eGFP-F-T461I in OCC even in the absence of known entry receptor (Figure 5B–D) while the MeV-IC323-eGFP-F-wt needs the expression of SLAM in order to disseminate efficiently in the OCC (Figure 5A).

## 10. MeV Dissemination in the CNS

In SSPE brain tissue, extracellular MeV has not been detected, suggesting that neuron-to-neuron viral dissemination can occur without released infectious viral particle [182]. MeV spread in mice and rat neurons is based on cell-to-cell contact [139,192,193]. The functional analysis of hyperfusogenic MeV bearing a mutated F protein T461I confirmed this theory by being able to disseminate exclusively from cell-to-cell in human primary neurons [124,128]. The combination of mutations found in SSPE strains seems to enable viral fitness in the brain and neurovirulence [128]. Viruses with these mutations can spread in the brain of genetically modified mice [111].

It is suggested that MeV dissemination can be mediated by the microfusion at synaptic membranes [97,128]. In this theory, the F protein may interact with Neurokinin-1, the receptor of the P substance [96,139] (Figure 5B). This interaction would lead to the formation of a fusion micropore, allowing viral RNP to pass disseminate through neurons without the need of neither budding nor other receptor engagement. This could also explain the lack of syncytia formation in human primary neurons following infection with hyperfusogenic MeV forms. It has also been hypothesized that some supporting cells of myelinated nerves could block cell-to-cell contact between neurons and trans-synaptic spread in the brain could be the only way to allow viral dissemination [38].

It is strongly suggested that neurovirulent MeV strains are using a third receptor or co-receptor yet unknown. Nevertheless, the hypothesis that single or combination of mutations would be sufficient to confer adaptation in brain tissues for infection and dissemination without the engagement of any receptor is also relevant.

### Models to Study the Dissemination?

Neuronal cell lines such as human cells NT2, human astrocytoma cells, or mouse neuroblastoma cells were also used, but their relevance remains difficult to appreciate when considering the important variation of behavior of cells out of their tissue context [31,97,192,194,195,196]. Primary neurons or neural polycultures were also often used [97] but are poorly representative of the neural population in human brain. In many studies, these cultures have been useful to investigate both intra and inter-neuronal spread of MeV [96], especially because they can be made from the brain of any transgenic mice.

The recently developed three-dimensional (3D) human brain organoid model has a high potential in order to investigate viral dissemination and evolution in the brain. The 3D brain organoids are generated from human pluripotent stem cells or human embryonic stem cells. This more ethical model offers a unique opportunity in generating relevant data that could be transposed faithfully to brain infection in humans [197]. Human brain organoids still require further development in order to overcome the lack of microglia and vascularization, but also their high cost and variability of the system [198]. However, to date, this model can be very useful in combination with ex vivo models, especially to test the efficacy of inhibitors in the context of brain infection, to follow viral dissemination and highlight the emergence of mutations.

## 11. Treatments

### 11.1. Symptomatic Treatment

Very few treatments are available against MeV infection and there is no therapeutic treatment for MeV-related encephalitis. The very first therapy administered after initial signs of infection are mainly supportive and focus on symptoms such as fever, dehydration, and diarrhea. Then, most of the treatments are generally dedicated to prevent or to cure from super infection such as pneumonia, often observed in infected patients. Antibiotics are commonly used to treat the complications related to bacterial superinfection [199].

### 11.2. Treatment Based on the Enhancement of Immune Response

In order to enhance the immune response, ribavirin, interferon alpha (IFN-α), and immune serum globulin can also be used clinically to treat MeV infection.

#### 11.2.1. Immune Serum Globulin

From the 1940s, intramuscular injection of immune serum globulin was reported to confer up to 79% protection to unvaccinated patients having close contact with measles infected patients [200]. More recently, effectiveness of immune serum globulin as post-exposure prophylaxis was estimated from 50% to 69% during the 2014 measles outbreak in British Columbia in Canada. However, this estimation is highly controversial because many other factors could have contributed to prevent the appearance of the disease. Indeed, the potential pre-exposure immune status as well as the unknown exposure intensity and timing make the effectiveness of the immune serum globulin very difficult to quantify [201]. Moreover, the level of measles-specific antibodies has been shown to be lower when induced by the vaccine compared to the acquisition from a wild type measles infection [202]. This led to the necessity to increase the doses of immune serum globulin in order to maintain a protective level of measles antibodies [201]. However, as mentioned in paragraph 7.3, SSPE seems to develop mainly when the exposure to MeV occurs during the first years of age before the immune system is completely mature and when maternal antibodies are still lasting [17]. Additionally, administration of immunoglobulin may have led to SSPE cases [203] and the use of MeV-specific antibodies to treat rodents after infection via intracerebral route led to persistency of MeV infection and encephalitis [204,205,206]. Thus, the use of immunoglobulins to treat measles infection should be very carefully thought before introduction in therapies and would greatly benefit from the combination with other antivirals acting at different levels of the viral replication cycle in order to cure the infection instead of inducing persistency.

#### 11.2.2. Ribavirin, IFN-α, Isoprinosine

Ribavirin is an antiviral drug with a broad antiviral activity, initially used for treatment of HCV [207]. It is a nucleic acid analog derived from guanosine and its main antiviral activity shown in vivo is its incorporation as a mutagenic nucleoside by the viral RNA polymerase [208]. The use of ribavirin and immune serum globulin seems to decrease respiratory symptoms in MeV-infected patients [209] but to date there is no standard protocol and doses recommended to treat patients.

IFN-α, ribavirin, and inosine pranobex are also used for SSPE treatment, with relative long-term effectiveness [210]. Many clinical reports show that Ribavirin can decrease measles antibody titers in cerebrospinal fluid (CSF) of SSPE patients and improve neurologic symptoms without side effects [211,212], especially when combined with IFN-α. In rare cases, long term IFN-α treatment stabilizes clinical symptoms of SSPE patients for years [213]. A recent study suggests also that continuous intraventricular administration of ribavirin and interferon-α in CSF by using a subcutaneous infusion pump, combined with oral administration of inosine pranobex, could limit the progression of SSPE [214]. Intrathecal IFN-α treatment combined with oral isoprinosine could also be effective to treat SSPE patients and is the most common treatment used nowadays [215,216]. Isoprinosine is a derivative of inosine and aims at blocking viral replication, probably through an immunoregulatory activity. Again, these treatments have rarely been shown to recover loss of function but they can stabilize the disease for several years [98,213,217]. Despite the benefits of IFN-α treatments, its use can be associated with side effects and could lead to interferonopathies [218]. Alternatively, there is induction of IFNα/β in vivo with MeV infection. This induction is associated at least partially to the presence of defective interfering (DI) particles which are also reducing the viral replication by occupying the proteins from the replication machinery and may thus constitute helpful complementary tool for treatments [219,220].

#### 11.2.3. Vitamin A

Vitamin A deficiency is highly related to measles complications and the supplementation of vitamin A has been shown to decrease the morbidity and mortality related to MeV infection in children [59,60,61]. Vitamin A is also mainly used to prevent blindness due to MeV infection in children [72,221]. Thus, WHO recommends immediate vitamin A administration to MeV-infected children with two repeated doses of 200,000 IU especially as vitamin A deficiency is a public health problem [3,222,223,224,225]. Nevertheless, vitamin A is also encouraged to be given in all severe cases, regardless of the country or patient age [225]. In severe cases of measles, the combination of vitamin A with ribavirin treatment can also decrease the morbidity [226].

At the beginning of the infection, the innate immune response relies on the detection of PAMPs (pathogen-associated molecular pattern) by pathogens recognition receptors (PRR) such as the RIG-I like Receptors (RLRs) in the cytoplasm [227]. This pathway allows the synthesis and the secretion of type-I interferon. Among the RLRs recognizing the double stranded RNA patterns for activation of the type I interferon response, RIG-I (Retinoic acid-inducible gene I) is activated by several RNA viruses including MeV [228,229,230].

Retinoic acid is a metabolic product of vitamin A (retinol) that inhibits MeV replication in vitro via a retinoid nuclear receptor-dependent pathway [231] and a type I interferon (IFN)-dependent mechanism [232].

The mechanism of action of vitamin A as an antiviral still needs to be better understood. Nevertheless, RIG-I is required for MeV inhibition by retinoids [233], suggesting an implication of RIG-I in the efficacy of vitamin A treatment.

#### 11.2.4. Interferon-Stimulating Genes (ISGs) and Other Treatments

The antiviral response is mediated by the interferon-stimulated genes (ISGs) that lead to the cell-intrinsic immunity. Recently, the overexpression of the bone marrow stromal antigen 2 proteins, also called BST2, Tetherin, or CD317, have been shown to inhibit Morbilliviruses cell to cell fusion in vitro by targeting the H protein [234]. In addition, the interferon-inducible transmembrane protein 1 (IFTIM1) has been shown to inhibit infection by several RNA viruses *in vitro*. While MeV enters via the plasma membrane, the effect of IFTIM1 on MeV replication is low compared to other *paramyxoviruses* such as the respiratory syncytial virus (RSV) but might be of interest in combination with other treatments [235].

Numerous other treatments, such as immunomodulators, carbamazepineamantadine, steroids, cimetidine, and plasmapheresis have been tested to treat SSPE but their efficacy seems to be case-dependent and need to be confirmed [216,217]. In addition, several alternative inhibitors such as antisense molecules, adenosine, and guanosine nucleosides, including ring-expanded “fat” nucleoside analogues, brassinosteroids, coumarins, modulators of cholesterol synthesis, and a variety of natural products have been investigated on MeV-infected patients. All these inhibitors showed relative efficacy or toxicity in vitro and in vivo and remain to be improved [236]. Among patients who received two doses of vaccine after initial infection some developed SSPE suggesting that the vaccine may not act as a therapeutic cure and prevent from encephalitis in this particular case [237].

### 11.3. Transcription/Replication Inhibitors

In order to inhibit the MeV growth, a strategy is to silence mRNAs encoding one of the key polymerase complex, namely N, P or L using small interfering RNAs (siRNAs) or shRNAs, as synthetic oligonucleotides, encoded by plasmids, or transduced using lentiviral vectors. siRNAs targeting the mRNA of either L [238] or N [239] or P [240], or the three in combination [241] have shown their efficiency in preventing virus growth over few days without cytotoxic effect. However, MeV finally escapes the silencing even in cells that constitutively express the siRNAs without acquiring any mutation even those that could disrupt the siRNA target sequence [240]. This likely reflects the remarkable long half-life of the polymerase brought by the incoming virus particles that last at least over 24 h [242,243] and the saturation of the siRNA linked to the continuous viral mRNA synthesis by the incoming polymerases.

As mentioned in Section 2, MeV P interacts with L protein. Although this interaction is independent of heat shock proteins such as the heat shock protein 90 (HSP90), both MeV P and HSP90 are necessary to fold and stabilize functional MeV L proteins able to enter the polymerase complex [243]. This transient requirement of HSP90 constitutes a potential target for transcription inhibition. Indeed, Geldanamycin and derivates such as 17-DMAG blocking HSP90 chaperon activity by entering its ATP pocket. These compounds showed the ability to block the viral transcription in preventive and post infection treatment in vitro and ex vivo in organotypic brain cultures [243]. Moreover, it is unlikely that a HSP90 inhibitor leads to the emergence of escape mutant virus [244]. While already used in cancer treatment, antivirals directly targeting the chaperon activity of HSP90 might be too toxic for human application. Nevertheless, molecules interfering between HSP90 and L and thus its functional folding are of interest for antiviral cure development.

Nucleoside analogs such as Remdesivir (GS-5734) and R1479 exhibit a broad spectrum activity against *paramyxoviruses* infections, including MeV [245]. Briefly, in cells, Remdesivir is metabolically converted to active nucleoside triphosphate. Obtained metabolite specifically inhibits several polymerases from different *Mononegavirales* such as Filoviruses and Henipaviruses, but not host polymerases. Recently, Remdesivir has been shown to inhibit Nipah virus polymerase activity by delaying the chain termination synthesis, notably in vivo in the African green monkey model [245,246]. Based on the huge conservation of the polymerases among *Mononegavirales*, there is a high probability that Remdesivir may also inhibit measles virus polymerase activity. Interestingly, pharmakokinetic studies performed in non-human primates showed high and persistent levels of the active metabolite in peripheral blood mononuclear cells (PBMCs) mainly targeted by wt MeV during the early stages of the pathogenesis [247]. Additionally, Remdesivir and subsequent active nucleoside seem to be able to reach the brain and may thus also inhibit CNS adapted variants of MeV observed in MIBE and SSPE cases.

Finally, the compound 16677 (1-methyl-3-trifluoromethyl-5-pyrazolecarboxylic acid) has been described as a non-nucleoside inhibitor of the RNA-dependent RNA polymerase complex activity [248]. The way this compound interacts with the replication machinery as well as the emergence of resistant variants remain poorly documented. Nevertheless, when tested in combination with an entry inhibitor increasing the stability of the fusion protein, the use of such replication inhibitor offered a high potential as a specific treatment against MeV. More recently, the same group has shown that compound AS-136A, analog to 16677, was able to block viral RNA synthesis by targeting L protein. This compound has also been associated to three candidates’ hotspots of mutation increasing the knowledge of L sequence adaptation [249]. In order to face its poor solubility in water, known to influence the antiviral activity, structure-activity relationship investigations were driven to discover analogs which could be used in vivo and resulted in the generation of orally bioavailable compound 2O (ERDRP-00519) more potent and aqueous soluble than former generation [250]. As the former candidates, this antiviral remains quite cytotoxic but could be particularly efficient in combination with fusion inhibitors or antiviral immune response activators.

### 11.4. Inhibitors of MeV Fusion and Entry

As mentioned in Section 2, the first step of the infection relies on entry of the virus into its target cell. Briefly, H protein engages entry receptor and triggers F protein. F exposes its highly hydrophobic fusion peptide which inserts into host cell plasma membrane. This transient intermediate stage is highly unstable. Consequently, F undergoes serial conformational changes leading to the interaction between the two heptad repeat domains that brings the two membranes close enough to merge and form the fusion pore. The viral RNP can thus enter in the cell host cytoplasm. In order to prevent viral entry, the main target is to block fusion of the virus. Blocking the interaction with the receptor or F serial conformational changes are the two mainly considered possibilities.

The receptor binding site of MeV H is considered as a potential neutralizing target. Indeed, the insertion of any compound in the H pocket responsible for the binding to the receptor could either prevent from the virus attachment to the host cell or pre-trigger the F protein leading to fusion dead viral particles. Several neutralizing antibodies targeting the H protein have been proposed mainly resulting in the emergence of resistant mutants not anymore able to bind either SLAM or nectin-4 [251]. While this loss of function should not exist in the wild, the question of the ability of such variants to invade the CNS which does not express SLAM or nectin-4 receptor under this selective pressure still needs to be investigated. More recently, neutralizing antibody-derived molecules such as single chain variable fragments targeting the H protein represent a major advance in the field of therapeutics design [252]. As for the corresponding neutralizing antibodies, the ability of such molecules to penetrate the brain parenchyma and to block hyperfusogenic variants depending less on the receptor engagement as those commonly observed in CNS infection has never been tested.

As described in Section 10, Neurokinin-1 has been shown to be a potential receptor for MeV F. As an antagonist of Neurokinin-1, Aprepitant has been shown to drastically limit the viral dissemination of vaccine strain in the brain of CD46+/RAG-2ko mice [96].

The fusion inhibitor peptide (FIP), Z-D-Phe-L-Phe Gly that is a small hydrophobic peptide and other small molecules such as AS-48 or 3G (an analogue of AS-48) can block the membrane fusion in vitro [125,126,253]. These inhibitors are known to stabilize the prefusion state of the F protein. Nevertheless the use of these inhibitors leads to the emergence of mutations in the HRC of the F that can evade their efficacy leading to the selection of MeV hyperfusogenic variants [254].

**Figure 6:**
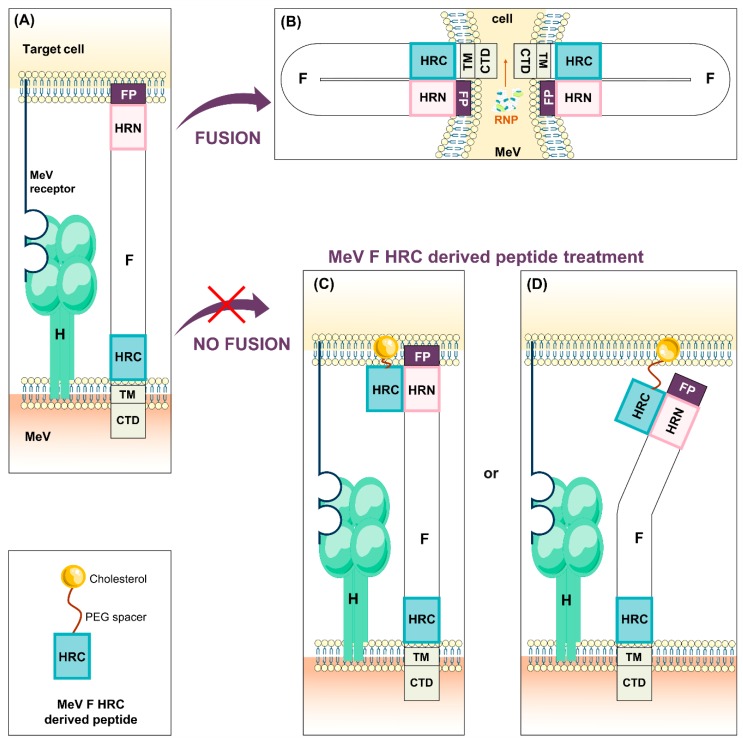
MeV F heptad repeats at the C terminal domain (HRC)-derived peptide. Following its engagement with any MeV receptor, H triggers F which inserts its fusion peptide in the host membrane (**A**). Then, F undergoes serial conformational changes to reach its post fusion state, bringing the two membranes close enough to form a fusion pore (**B**). MeV F HRC-derived peptides interact with MeV F HRN and catch the intermediate states of MeV F to block the fusion, regardless of the insertion of the fusion peptide in the host membrane (**C**,**D**).

In contrast, HRC-derived peptides, aim at blocking the fusion by capturing the F protein in the post-triggering state and freezing the fusion process at an early stage (Figure 6A–D). The so-called HRC4 peptide is a MeV F HRC-derived dimeric peptide that interacts with the HRN domain during the structural transition of F (Figure 6C,D). Briefly, HRC4 peptide is a dimer constituted of the HRC derived peptide linked with two chains of PEG that acts as a spacer each conjugated with a molecule of cholesterol. The cholesterol allows the fusion peptide to anchor into the host membrane and thus increases the antiviral potency of the HRC-derived peptide by two logs [255]. The HRC-derived peptides conjugated to cholesterol as well as tocopherol have shown high efficacy in vitro, ex vivo and in vivo, even in the context of the CNS infection by crossing the blood-brain barrier [29,42,146]. Notably, dissemination of viruses bearing the L454W mutation in F can be efficiently blocked in vitro and in vivo by F HRC-derived fusion inhibitors, regardless of the presence of SLAM [42]. To date, these fusion inhibitors are the only system already tested against both the wt and hyperfusogenic variants observed in CNS infection, and figure thus among the priority candidates for preclinical studies, to test alone and in combination with treatments targeting other viral functions.

## 12. Conclusions

A better understanding of MeV CNS invasion remains a priority in the field of MeV studies, especially because of the recent re-emergences of measles and the increasing number of associated fatal encephalitis [44,256]. While the vaccine remains the most efficient prevention against MeV infection, the decreasing coverage combined to the increasing number of immunocompromised people difficult to vaccinate confirm the necessity to develop efficient antiviral strategies.

To date, the emergence of the mutations observed in the brain of SSPE or MIBE patients is still poorly understood. These mutations could have emerged through an adaptation to the brain, leading to SSPE or MIBE, or through a selection of pre-existing mutations as a polymorphism among the circulating strains. Since MIBE only concerns immunocompromised patients and occurs usually very shortly after the primary MeV infection, one could speculate that it is more likely that a minor population of MeV, bearing the mutations that allow the virus grow in a neural context, gets selected and take the advantage of this immunological status for further propagation in the brain. Regardless of the type of encephalitis or MeV variant invading the brain, the high mortality rate associated to measles virus CNS complication highlight the requirement to validate antiviral molecules against these variants.

While the number of tested potential antiviral therapeutics keeps growing, a single molecule or treatment capable to block the major viral cycle steps is still not available. Ultimately, a combination of the treatments that could block the viral entry, the dissemination, the replication, and stimulate the immune system seems to be the most promising solution to prevent and cure MeV systemic infection and will be even more critical for the treatment of CNS infection.

## Figures and Tables

**Table 1:** Small animal models used to study MeV infection. IFNAR stands for interferon alpha/beta receptor, Rag for recombination activating gene, and TLR for Toll-like receptor.

Animal	Purpose	Route of Infection
MICE		
NSE-CD46: Expression of BC1 isoform of human CD46 under the control of the neuron-specific enolase (NSE) [159,160] YAC-CD46: Similar expression level and location than in human [159] CD46 [143,161,162] CD46-IFNAR^−/−^ [163]	Vaccinal MeV behavior in the brain. Ability to disseminate in the brain. Pathogenesis of MeV infection in the CNS. Permissiveness. Immune response.	Intranasally (i.n.)Intracranially (i.c.)
SLAM: Ubiquitous expression [144] SLAM: Dendritic cell expression only [164,165] CD46/TLR induced CD150 [166] CD46/TLR induced CD150-IFNAR^−/−^	Innate immune response Spreading and pathogenicity of Edmonston and wild-type Ichinose (IC) strains	i.n. i.c.
SLAM IFNAR^−/−^ [167] (Figure in Section 9.3) IFNAR^−/−^ [42,168] (Figure in Section 9.3)	Tropism Dissemination within CNS	Intraperitoneal (i.p.) i.n.
SLAM^+/+^/Stat 1^−/−^: Same expression level than human [169]	Innate immune response	i.p. i.n.
CD46 IFN-α/βKO [163]	Induction of MeV encephalitis with Edmonston	i.c.
CD46 RagKO [96,103]	Study of the establishment of SSPE Role of the immunosuppression in the MeV persistence Drug testing	Multiple
CD46 Neurokinin-1 KO [96]	Trans-synaptic viral dissemination	i.c.
C57BL/6 [170] (Figure in Section 9.3)	Viral persistence Tropism	i.c.
RAT		
Lewis rat [136,139,171]	Tropism	i.c.
Cotton rat (Sigmodon hispidus) [12,172,173]	Treatment development. Respiratory infection MeV induced immunosuppression CNS infection Immune suppression	i.n.
CD46 Sprague-Dawley rat [174]	permissiveness	Multiple
Brown Norway rat [175]	Immune response in MeV associated neurologic disease	i.c.
HAMSTER		
Syrian Golden Hamster [123] (Figure in Section 9.3)	Tropism Dissemination and invasion by mutated viruses	i.c.
FERRET		
Ferret [97,155,176,177]	To mimic SSPE Transmission Pathogenesis of CDV infection to model MeV infection	i.c. i.n.
